# Effects of meteorological and land surface modeling uncertainty on errors in winegrape ET calculated with SIMS

**DOI:** 10.1007/s00271-022-00808-9

**Published:** 2022-08-13

**Authors:** Conor T. Doherty, Lee F. Johnson, John Volk, Meagan S. Mauter, Nicolas Bambach, Andrew J. McElrone, Joseph G. Alfieri, Lawrence E. Hipps, John H. Prueger, Sebastian J. Castro, Maria Mar Alsina, William P. Kustas, Forrest S. Melton

**Affiliations:** 1 Department of Civil and Environmental Engineering, Stanford University, Stanford, CA USA; 2 Division of Science & Environmental Policy, California State University, Monterey Bay, Seaside, CA USA; 3 Earth Science Division, NASA Ames Research Center, Moffett Field, CA USA; 4 Hydrologic Sciences Division, Desert Research Institute, Reno, NV USA; 5 Department of Land, Air and Water Resources, University of California, Davis, CA USA; 6 Crops Pathology and Genetics Research Unit, USDA-ARS, Davis, CA USA; 7 Department of Viticulture and Enology, University of California, Davis, CA USA; 8 Hydrology and Remote Sensing Laboratory, USDA-ARS, Beltsville, MD USA; 9 Department of Plants Soils and Climate, Utah State University, Logan, UT USA; 10 National Laboratory for Agriculture and the Environment, USDA-ARS, Ames, IA USA; 11 E and J Gallo Winegrowing Research, Modesto, CA USA

## Abstract

Characterization of model errors is important when applying satellite-driven evapotranspiration (ET) models to water resource management problems. This study examines how uncertainty in meteorological forcing data and land surface modeling propagate through to errors in final ET data calculated using the Satellite Irrigation Management Support (SIMS) model, a computationally efficient ET model driven with satellite surface reflectance values. The model is applied to three instrumented winegrape vineyards over the 2017–2020 time period and the spatial and temporal variation in errors are analyzed. We illustrate how meteorological data inputs can introduce biases that vary in space and at seasonal timescales, but that can persist from year to year. We also observe that errors in SIMS estimates of land surface conductance can have a particularly strong dependence on time of year. Overall, meteorological inputs introduced RMSE of 0.33–0.65 mm/day (7–27%) across sites, while SIMS introduced RMSE of 0.55–0.83 mm/day (19–24%). The relative error contribution from meteorological inputs versus SIMS varied across sites; errors from SIMS were larger at one site, errors from meteorological inputs were larger at a second site, and the error contributions were of equal magnitude at the third site. The similar magnitude of error contributions is significant given that many satellite-driven ET models differ in their approaches to estimating land surface conductance, but often rely on similar or identical meteorological forcing data. The finding is particularly notable given that SIMS makes assumptions about the land surface (no soil evaporation or plant water stress) that do not always hold in practice. The results of this study show that improving SIMS by eliminating these assumptions would result in meteorological inputs dominating the error budget of the model on the whole. This finding underscores the need for further work on characterizing spatial uncertainty in the meteorological forcing of ET.

**Supplementary Information:**

The online version contains supplementary material available at 10.1007/s00271-022-00808-9.

## Introduction

Evapotranspiration (ET) is controlled by the present state of both the land surface and the near-surface meteorological conditions. Calculating ET using biophysical models requires information about the land surface, including the availability of water in soil or vegetation and the rate at which it can be conducted to the atmosphere. It also requires information about local meteorological conditions, like temperature and humidity, that determine evaporative demand. Incomplete information about local conditions necessitates estimating or making assumptions about the values of several ET forcing variables. Judging the accuracy and robustness of any ET estimation method requires examining the spatial and temporal dynamics of errors in model inputs and the impact of those errors on final ET estimates. Quantification of model errors is important when applying remote sensing-based ET estimation methods to water resource management problems (Foster et al. 2020).

This paper examines the contribution of meteorological and land surface data inputs to errors in ET estimates using the Satellite Irrigation Management Support (SIMS) model (Melton et al. 2012; Pereira et al. 2020) for three climatically distinct winegrape vineyards in California. SIMS is used for agricultural applications like irrigation scheduling, as well as for larger scale water resource management and assessment. The SIMS model is computationally efficient and provides ET estimates that use satellite data to account for the current crop growth stage, canopy extent and condition, and provide users with ET values that represent crop water requirements under well-watered conditions. These features make SIMS useful for irrigation scheduling and management. This analysis of the contribution of model inputs to the spatial and temporal distribution of model ET errors will be useful to practitioners who rely on SIMS ET estimates, and also for future improvement of the model. While this study focuses on errors in SIMS, some of the results will have implications for remote sensing-driven ET estimation more generally. There are many different approaches for estimating ET and many different parameterizations of the soil–vegetation–atmosphere system. However, any model must account for the fact that ET can be limited either by meteorological conditions (local evaporative demand) or by land surface conditions (availability of water and plant physiology). Therefore, any ET estimation method can be affected by uncertainty in both meteorological and land surface data inputs, which are the focus of this analysis.

To account for meteorological forcing of ET, SIMS relies on the ASCE Penman–Monteith grass reference ET (ETo) (Allen et al. 2005; Walter et al. 2000) calculated using meteorological data collected by the California Irrigation Management System (CIMIS). CIMIS uses the grass reference as the standard for California and CIMIS weather stations are installed over grass surfaces. ETo is commonly used to account for atmospheric evaporative demand and radiative forcing of ET for agricultural applications like irrigation scheduling (Allen et al. 1998). While different models parametrize atmospheric and radiative forcing of ET differently, these calculations all rely on measurements or estimates of basic physical variables like air temperature, humidity, windspeed, and net radiation. Errors in these common inputs can have similar impacts on different models of potential ET. A challenge in real-world applications is accurately estimating the values of these physical variables at locations in between weather stations. One aim of this study is to characterize the errors in overall ET estimates resulting from uncertainty related to meteorological forcing.

While there have been multiple studies that have characterized the errors of satellite-driven energy balance models over vineyard sites (Knipper et al. 2020, 2019; Semmens et al. 2016; Carrasco-Benavides et al. 2012), few studies have evaluated reflectance-based approaches for calculation of ET over winegrape vineyards. A notable exception is an approach using Sentinel-2 satellite shortwave infrared (SWIR) bands that are found to be responsive to crop water status and soil moisture and have recently have been incorporated into the Shuttleworth–Wallace modeling scheme (Shuttleworth and Wallace, 1985) for estimating vineyard ET (D’Urso et al. 2021). SIMS is a reflectance-based model that implements portions of the FAO-56 dual crop coefficient model (Allen et al. 1998), a widely used parameterization of the soil–vegetation–atmosphere system for ET estimation in irrigated agriculture. SIMS operationalizes this model using a combination of remotely sensed vegetation measurements and spatially resolved crop type information. The model can be run using surface reflectance measurements available from multiple satellites and uses the density coefficient approach developed by Allen and Pereira (2009) to derive basal crop coefficients from satellite observations of the crop canopy. SIMS has been shown to produce useful ET estimates in practice, particularly for irrigated agriculture in the Western United States (Melton et al. 2021; Pereira et al. 2020).

SIMS calculates crop ET (ETc) as the product of two values, ETo and a basal crop coefficient (Kcb): ETo * Kcb = ETc. Kcb accounts for both the availability of water and the ability of the crop to conduct that water to the atmosphere. In this study, we focus on attributing errors to uncertainty in meteorological versus land surface components of the model. In practice, ETo and Kcb are themselves computed using many input variables, the values of which must be measured, estimated, or assumed. Within the SIMS model, inputs to Kcb are computed using a combination of multispectral remotely sensed measurements (canopy cover fraction) as well as prior information or assumptions about vegetation type and stomatal control (Melton et al. 2012; Pereira et al. 2020). Because the assumptions of SIMS are clear and physically interpretable, we can make stronger claims about the representativeness of the error characterization.

Understanding the dynamics and impacts of errors in model input data will be useful to practitioners who use SIMS and related satellite-driven or FAO-56 crop coefficient models for applications including irrigation scheduling and water resource monitoring. In addition, error propagation observed in SIMS is likely to be representative of that in other classes of ET models because of similarities in the treatment of meteorological forcing terms. For example, the impact of local versus regional meteorological inputs into the reflectance-based ET model of D’Urso et al (2021) indicates significant errors can result using non-representative meteorological forcing (Bhattarai et al., 2022). Finally, SIMS uses a relatively simple approach to estimate land surface conductance, one which has a clear physical interpretation. As such, SIMS offers a straightforward basis for assessing the relative contribution of different data inputs to errors in final ET estimates. In principle, reflectance-based approaches can be improved upon by incorporating additional remotely sensed or in situ measurements like soil moisture or land surface temperature. Results using SIMS will give an indication of how much error reduction would be possible using a more complex land surface model that can represent ET variation arising from such factors.

The first part of this study compares the impact of using ETo computed from in situ measurements versus ETo estimates from a gridded data product. For the comparison, ETo estimates are derived from measurements at the weather stations comprising the California Irrigation Management Information System (CIMIS). The California Department of Water Resources (CDWR) distributes a gridded ETo product ("Spatial CIMIS") that combines geostationary satellite imagery with interpolated meteorological variables between weather stations (Hart et al. 2009). While useful in the absence of in situ measurements, all spatially interpolated weather data products necessarily introduce errors due to unknown spatial variation (not captured by geostatistical models) and representativeness error when downscaling from multi-kilometer grid cells to field scale. Our analysis will help practitioners understand the characteristics and impact of errors introduced using spatially interpolated ETo values when estimating ET and there is no weather station nearby.

The second part of the study compares SIMS crop coefficient estimates with the ground-based measurements of fraction of grass reference ET (EToF), computed as the ratio of actual ET measured at each flux tower and ETo. In particular, we examine estimates of the basal crop coefficient (Kcb), which SIMS computes as a function of normalized difference vegetation index (NDVI) and fractional cover data following the density coefficient approach (Allen and Pereira 2009; Pereira et al. 2020). Kcb is expected to correspond to the EToF in a field or vineyard where the crop is well watered (transpiration is not water limited) and the exposed soil surface is dry. The overall dual crop coefficient Kc also includes the effects of soil evaporation (Ke) and water stress (Ks) (Allen et al. 1998), but estimating these contributions requires knowing soil water content in the evaporable and root zones, respectively. While the soil system can be modeled, in general it is difficult to remotely determine irrigation schedules or volumes without direct input from a user with knowledge of irrigation applications. For simplicity, we will still refer to the difference between SIMS Kcb and ground-based EToF as error despite the fact that Kcb * ETo is technically not an estimate of ETa in general, though it is commonly used as a proxy in practice. Observed differences between SIMS Kcb and ground-based EToF can be explained by one or more of the following: (a) SIMS estimate of basal crop coefficient, Kcb, is different from the true (idealized) Kcb, (b) evaporation is occurring from a wet soil surface (Ke > 0, thus Kcb < Kc), or (c) transpiration is water limited (Ks < 1, thus Kcb > Kc). While we cannot definitively disaggregate errors into these three sources, we can assess their relative magnitude and variability. One goal of this analysis is to characterize the errors that result when using SIMS Kcb as estimates of EToF. This analysis provides insight for practitioners who rely on crop coefficient estimates for irrigation scheduling and can also inform future development of the SIMS model.

### Methods

**Fig. 1:**
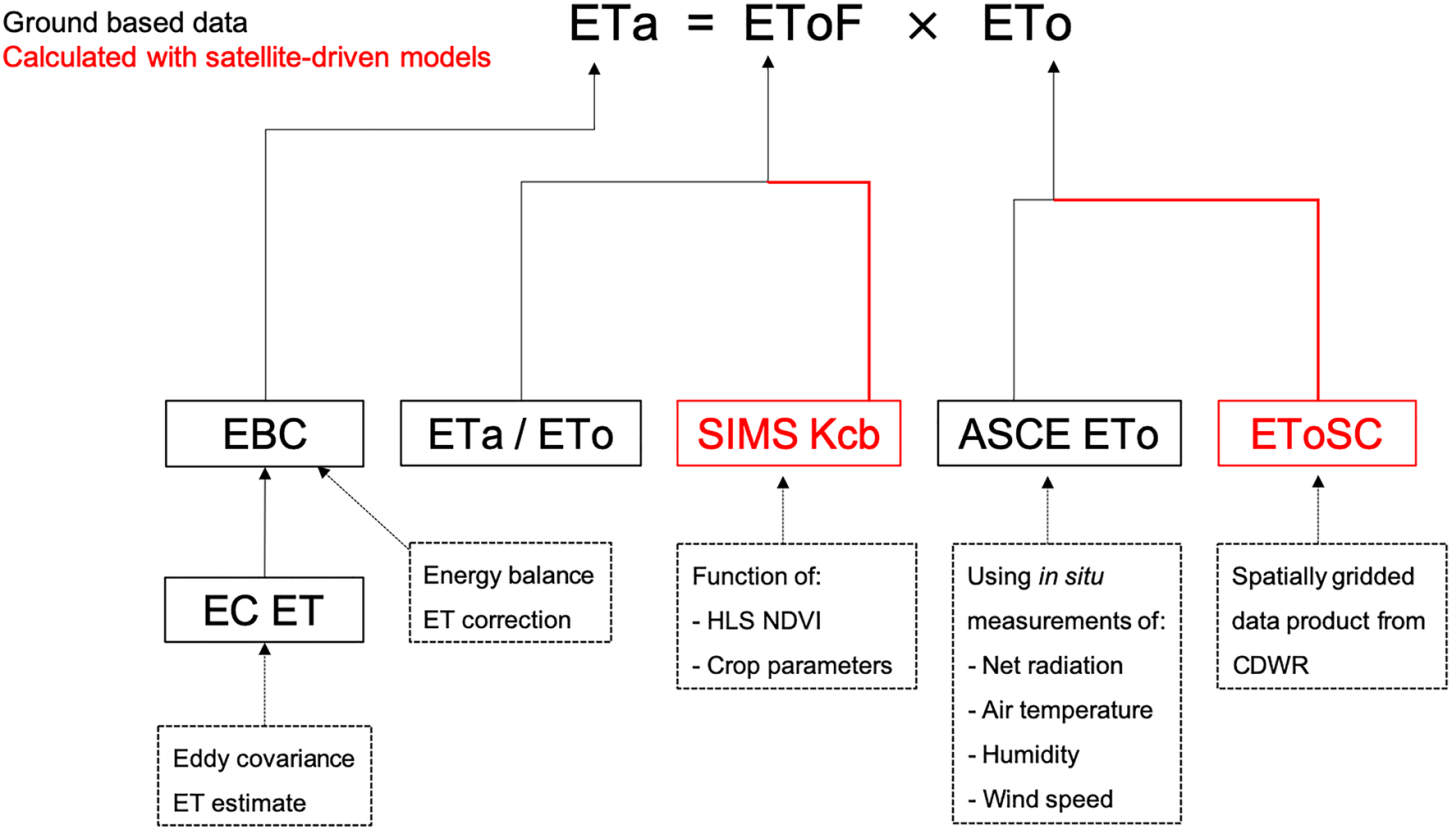
Schematic of model inputs, data sources and processing steps. The black solid border boxes correspond to values calculated using in situ measurements that are taken as "ground truth" for the purpose of analysis. The red boxes correspond to values that are calculated using a model, whose contribution to overall ET errors are being studied. The boxes with dashed line borders provide additional detail on inputs

In the following sections, we describe the data and procedures used to produce actual ET (ETa) from in situ measurements, ASCE Penman–Monteith grass reference ET (ETo) from in situ measurements, Spatial CIMIS ETo data (EToSC), and SIMS Kcb data from remotely sensed measurements. The remaining variable used in comparisons, EToF, is computed as ETa / ETo. Table 1 summarizes all of the major variables included in this study. Figure 1 shows the high-level variables examined in this study and the data sources used to compute them.

**Table 1:** Definitions and descriptions of variables used in the study

Variable	Abbreviation	Data source	Calculation
Actual ET	ETa	In situ micro-meteorological instrumentation deployed on a flux tower within each vineyard	LE computed using eddy covariance measurements (Alfieri et al. 2019) with application of the energy balance closure method of Volk et al. (2021)
Reference ET	ETo	In situ meteorological instrumentation	Compute hourly ASCE ETo (Walter et al., 2000; Allen et al. 2005) from in situ measurements, sum hourly calculations to daily
Fraction of reference ET	EToF	In situ meteorological instrumentation	Computed as the ratio ETa/ETo
Spatial CIMIS gridded ETo	EToSC	CDWR Spatial CIMIS	Spatially gridded ETo estimates computed by interpolating meteorological variables between CIMIS weather stations and satellite-based computations of solar radiation
SIMS basal crop coefficient	Kcb	SIMS Kcb computed from surface reflectances from the Harmonized Landsat-Sentinel (HLS) dataset	Calculated using satellite retrievals of NDVI and crop-specific relationships between fractional cover, crop height, stomatal control, and potential transpiration

### In situ ET data

**Fig. 2:**
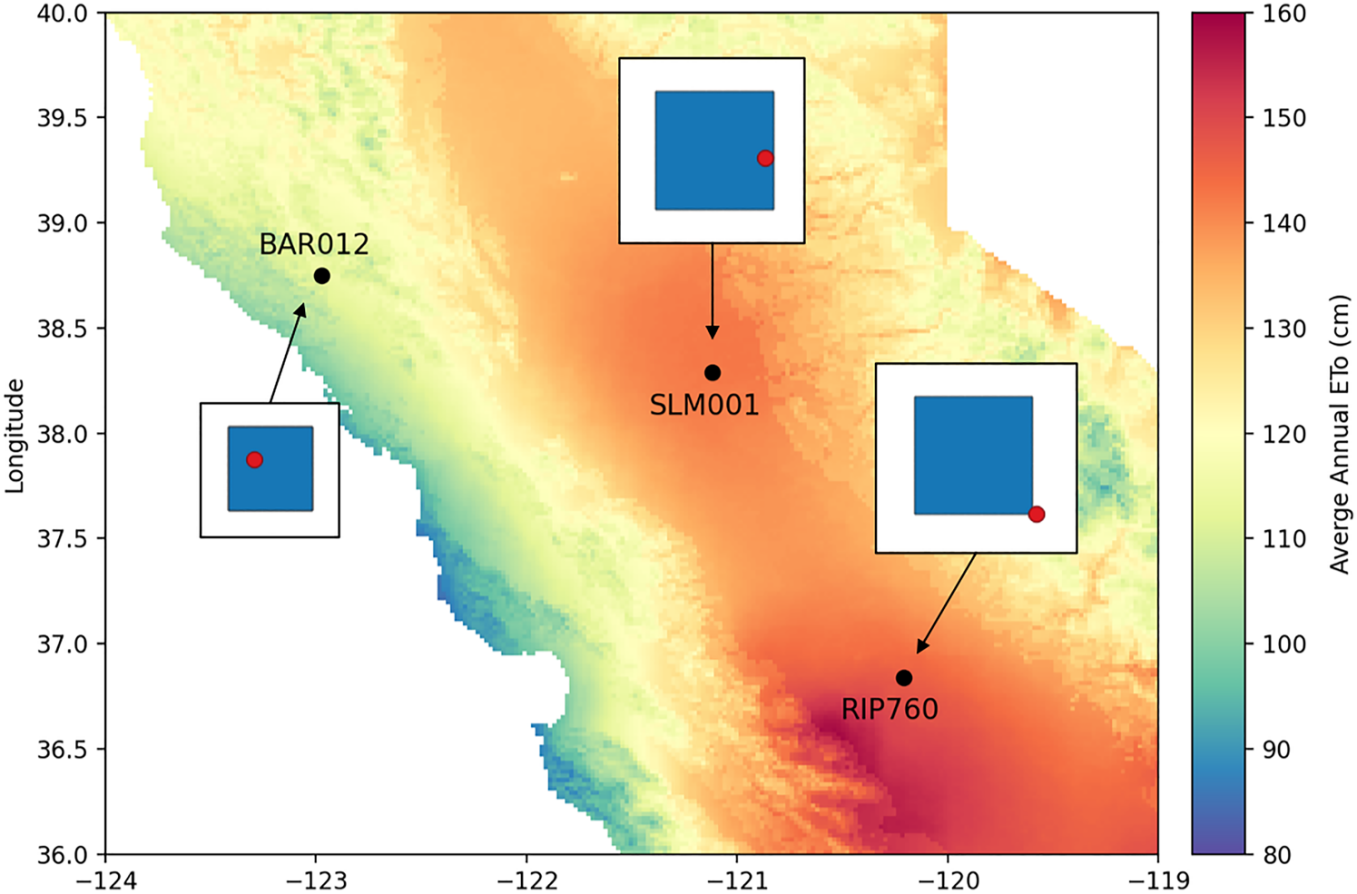
Vineyard locations and flux tower footprints. Black points show the locations of the vineyards in California. The colored background is the average annual EToSC for the duration of the study in cm, which illustrates climatic variation between the sites. For each site, the flux tower footprint (blue square) is shown relative to the position of the tower itself (red point). The footprints reflect average local wind conditions and were generated using the method described in Sect. 2.1. The footprints for SLM001 and RIP760 are 210 × 210 m, which corresponds to a 7 × 7 grid of 30 m pixels. The footprint for BAR012 is 150 × 150 m, which corresponds to a 5 × 5 grid of 30 m pixels

Eddy covariance flux data from three towers that are part of the Grape Remote Sensing Atmospheric Profile and Evapotranspiration eXperiment (GRAPEX) project (website) were used to calculate daily ET (Kustas et al. 2018). One tower is located in a Cabernet Sauvignon vineyard (BAR012) in the North Coast region and two in the Central Valley including a Pinot Noir (SLM001) and a Chardonnay vineyard (RIP760) (Fig. 2). The climate at BAR012 is substantially different from the climate at the Central Valley sites with estimated average annual ETo of 114 cm, 142 cm, and 147 cm at BAR012, SLM001, and RIP760, respectively. The three sites share similar instrumentation and are equipped to measure the four energy balance components: latent energy flux (LE), sensible heat flux (H), soil heat flux (G), and net radiation (Rn). Full details of the instrumentation used and data processing methods for the in situ measurements are provided in Alfieri et al. (2019) and briefly summarized here. Measurements of G were averaged from diagonal transects of five soil heat flux plates which span the vineyards and rows, and at each plate pairs of soil thermocouples and a soil moisture sensor are deployed to account for heat storage above the plate. A range of corrections and adjustments were made to raw flux measurements (LE and H); for example, filtering of anomalous high frequency (20 Hz) measurements using a Median Absolute Deviation approach (Mauder et al. 2013) and adjustment of fluxes using the Webb, Pearman, and Leuning density corrections (Webb et al. 1980). Micrometeorological and flux data were then aggregated over half-hourly periods. LE (W/m^2) fluxes are converted to ET (mm/day) using the known properties of water including its density and latent heat of vaporization.

For this analysis, half-hourly flux data were post-processed using the “flux-data-qaqc” Python package (Volk et al. 2021) to produce daily ET estimates that are corrected for energy imbalance. The method involves limited gap-filling of energy balance components, daily averaging of fluxes, and correction of daily LE and H fluxes using an approach based on the FLUXNET2015 dataset and ONEFlux flux processing methods (Pastorello et al. 2020). Briefly, the energy balance closure correction uses a centered sliding window (typically 15 days) of the daily energy balance ratio (LE + H / Rn—G) to partition the daily energy balance residual/error (Rn–G–LE–H) to LE and H such that the average corrected closure is adjusted towards a value of 1.0 over the sliding window time period. Observations that, after correction, produced an energy balance closure ratio below 0.75 were removed from the sample.

Estimates of remotely sensed ET from SIMS, which are produced at a spatial resolution of 30 × 30 m, were sampled around flux tower locations using a 7 × 7 pixel footprints (total size 210 × 210 m) at sites SLM001 and RIP760 and a 5 × 5 pixel footprint (total size 150 × 150 m) at site BAR012. Footprints were calculated following the methods described in (Melton et al. 2021). The footprints are shifted into the predominant upwind direction while maintaining overlap with the tower. Dominant wind speeds and directions were based on daytime (6:00–18:00 local time) windrose diagrams. The 150 × 150 m footprints were used at BAR012 because the larger footprint included areas outside of the vineyard boundary after accounting for the predominant upwind direction. SLM001 and particularly RIP760 have higher advection on average than BAR012 resulting in larger footprints and the flux towers being less centrally located within the footprint.

### ETo estimation from in situ measurements

ETo was calculated by the ASCE Penman–Monteith grass reference formulation (Walter et al. 2000; Allen et al. 2005). This is the same formulation of reference ET used in Spatial CIMIS. Hourly ETo (mm/hr) was derived using in situ measurements of the inputs to the ASCE hourly ETo equation, then hourly values were summed to get daily ETo (mm/day). Temperature and humidity were measured by a Campbell HMP45C probe, from which actual and saturation vapor pressure were computed (Alfieri et al. 2019). Elevation was known for each of the sites. Clear sky shortwave radiation was calculated using the method described in FAO-56 (Allen et al. 1998). Actual downwelling shortwave radiation was measured by an in situ radiometer. Average wind speed at 2 m above the canopy was computed using a sonic anemometer. While the actual land surface (winegrape vines growing on trellises) itself causes changes in wind behavior relative to the reference crop (short grass), we assumed that in situ wind measurements were still more representative of local variation than gridded estimates interpolated from the nearest agricultural weather stations.

### Spatial CIMIS ETo data product

Calculating ET over a spatially continuous area requires estimating the values of meteorological variables at unobserved locations (i.e., where there is no weather station nearby). It is a common practice to use estimates of meteorological variables generated by interpolation (to exactly fit measurement data where available) or smoothing (to minimize error while satisfying shape constraints) between weather stations over a 2D grid. Spatial CIMIS (Hart et al. 2009) is a daily, spatially gridded 2 km ETo data product distributed by the CDWR (website). This operational product is generated using multiple data smoothing methods for different meteorological variables, combined with hourly GOES visible-region satellite imagery (Diak and Gautier 1983; Gautier et al. 1980; NOAA Office of Satellite and Product Operations 1994).

### SIMS basal crop coefficient estimation

SIMS computes Kcb largely as a function of remotely sensed NDVI retrievals. For this study, we computed NDVI from Harmonized Landsat Sentinel-2 (HLS) surface reflectance (Claverie et al. 2018) red and near-infrared bands (website). The red band observations are from Landsat-8 OLI band 4 and Sentinel-2 band 4. The near-infrared band observations are from Landsat-8 OLI band 5 and Sentinel-2 band 8A. Observations were used from the following HLS tiles: T10SFH (SLM001), T10SEH and T10SDH (BAR012), T10SGF and T11SKA (RIP760). The NDVI rasters were clipped using the flux tower footprints described in Sect. 2.1. Eight-day composites were then computed for the multi-year time series by retaining the maximum value for each pixel over the compositing period. The number of clear observations range from 0 to 12 per composite period, varying by period and location based on satellite orbits and cloud cover. The composites were arranged in series and missing data (composite periods with no clear observations) were filled using linear interpolation. If the first (beginning of 2017) or last (end of 2020) composites had missing values, a fill value of 0.15 was used, which is a typical NDVI observed for bare-soil conditions (Johnson and Trout 2012). The NDVI composites were transformed to Kcb using the procedures described by Pereira et al. (2020) and parameter values reflecting the average stomatal control characteristics of winegrapes. The result of this processing pipeline was a series of Kcb rasters for each 8-day period. Kcb was assumed to be constant for each 8-day period. Daily average Kcb values for the average of the flux tower footprint were computed as the average of the pixel values within the footprint.

### Validation

This study compares ETa with ET estimates from (1) ground-based EToF and CIMIS ETo and (2) SIMS crop coefficient and ground-based ETo. For each of the comparisons, the following statistics were computed: (1) root mean square error (RMSE), which estimates the average magnitude of overall error, (2) bias, which is the average (signed) error, and (3) the unbiased root mean squared error (ubRMSE), which estimates the contribution of random error ("noise") to the overall error. These three statistics are related by the following relation: RMSE^2 = Bias^2 + ubRMSE^2 (Entekhabi et al. 2009). Correlation, which estimates the degree of linear relationship between variables irrespective of bias and differences in scale, was also computed for the ETo and Kcb/EToF comparisons. Correlation was not computed for the ETa/SIMS ET comparisons because ETa explicitly depends on ETo and EToF by assumption (i.e., would be computing Corr(X, X*Y)), so the statistic was not informative for our purposes and prone to misinterpretation. Across sites and years, there were days with a value of ETa but not ETo (e.g., temperature and humidity measurements missing) and days for which the opposite was true (e.g., sonic anemometer measurements missing). In the timeseries figures in Results, these days are plotted with one value or the other but are excluded from computation of summary statistics.

In addition to overall accuracy statistics, errors are summarized by site and by year using kernel density estimation (KDE) plots. KDE is similar to a histogram in that it summarizes the distribution of sample data. KDE is different from histograms in that it directly estimates the probability density function rather than simply counting samples that fall within non-overlapping bins. KDE uses a kernel function with a specified bandwidth that computes a local weighted average of the empirical density. The kernel function bandwidth is conceptually similar to bin width in a histogram in that it determines the scale at which observations are considered similar and controls the smoothness of the resulting density estimate. In this paper, the KDE plots are generated using the function kdeplot from the seaborn plotting library in Python (Waskom 2021). kdeplot computes the KDE using the gaussian_kde function from the Scipy statistics package (Virtanen et al. 2020). gaussian_kde automatically computes an appropriate bandwidth of the Gaussian kernel following the methods described by Scott (1992). The plotted curves are scaled to have cumulative density equal to one.

## Results

### Error from estimated ETo

**Fig. 3:**
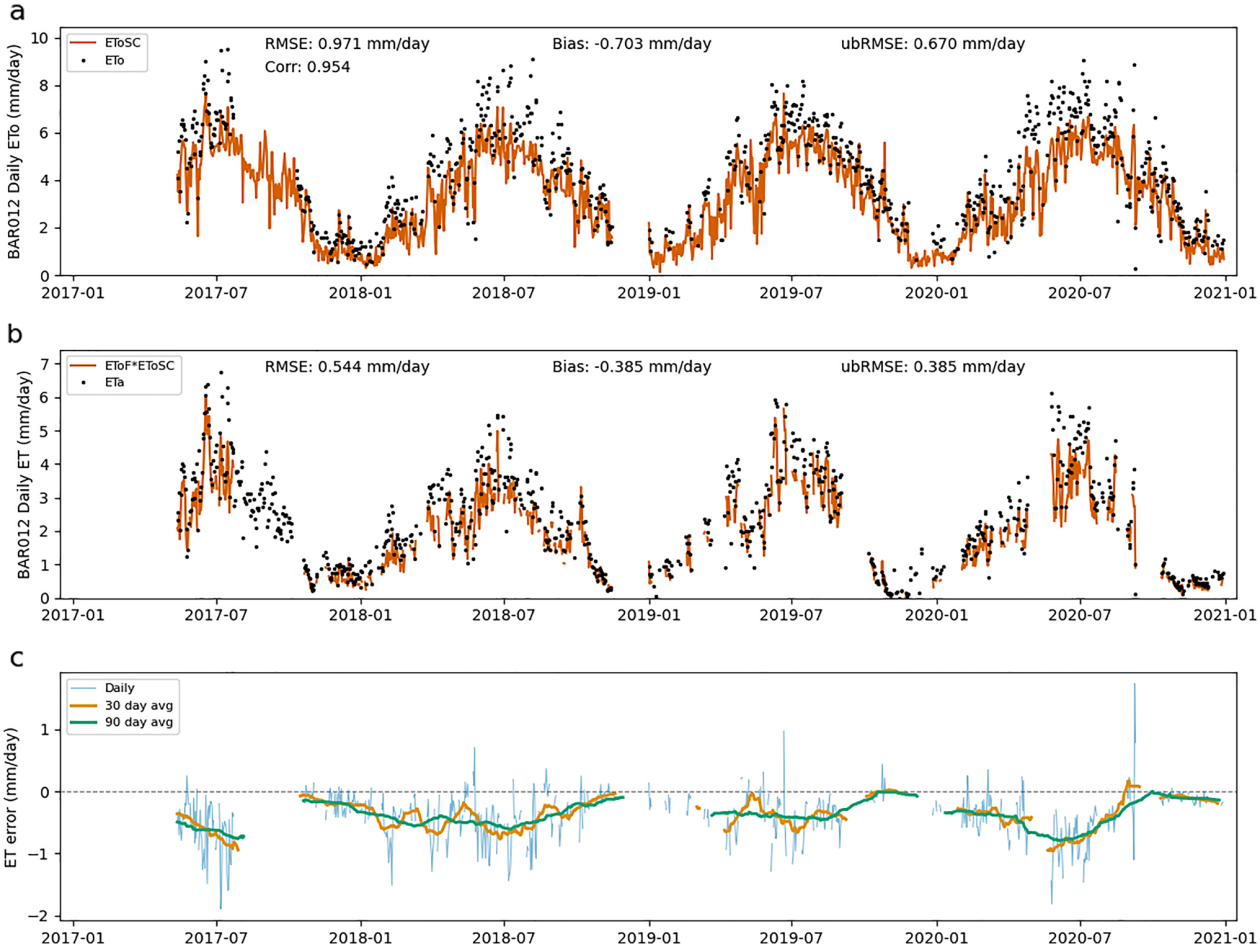
ETo errors at BAR012. Panel **a** shows EToSC versus ETo computed from in situ measurements at daily timesteps. Panel **b** shows ET computed as the product of EToSC and ground-based EToF versus ETa at daily timesteps. Panel **c** shows individual daily ET errors (Eta–EToSC*EToF) as well as averaged daily errors over 30- and 90 day windows

**Fig. 4:**
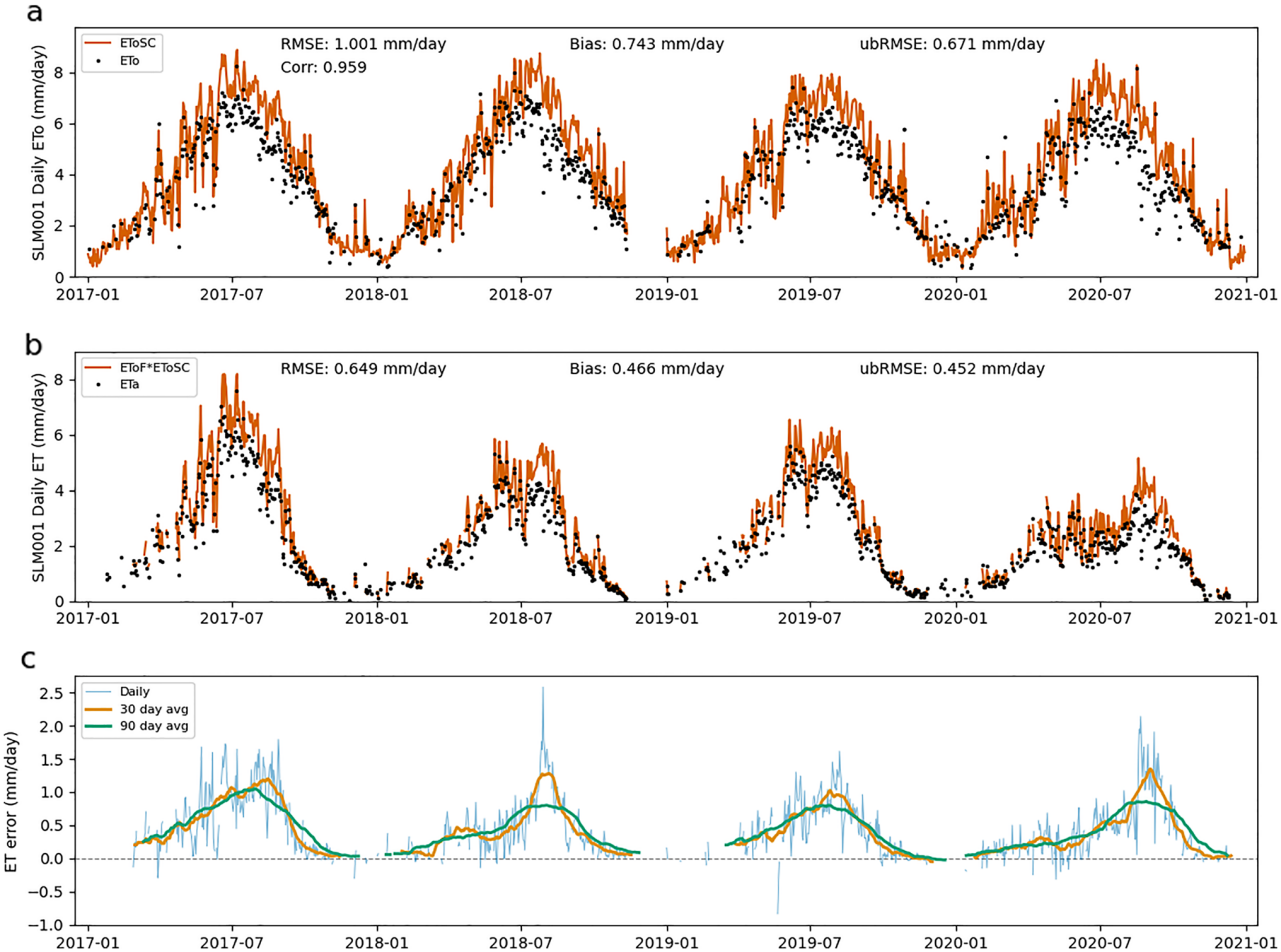
ETo errors at SLM001. Panel **a** shows EToSC versus ETo computed from in situ measurements at daily timesteps. Panel **b** shows ET computed as the product of EToSC and ground-based EToF versus ETa at daily timesteps. Panel **c** shows individual daily ET errors (Eta–EToSC*EToF) as well as averaged daily errors over 30- and 90 day windows

**Fig. 5:**
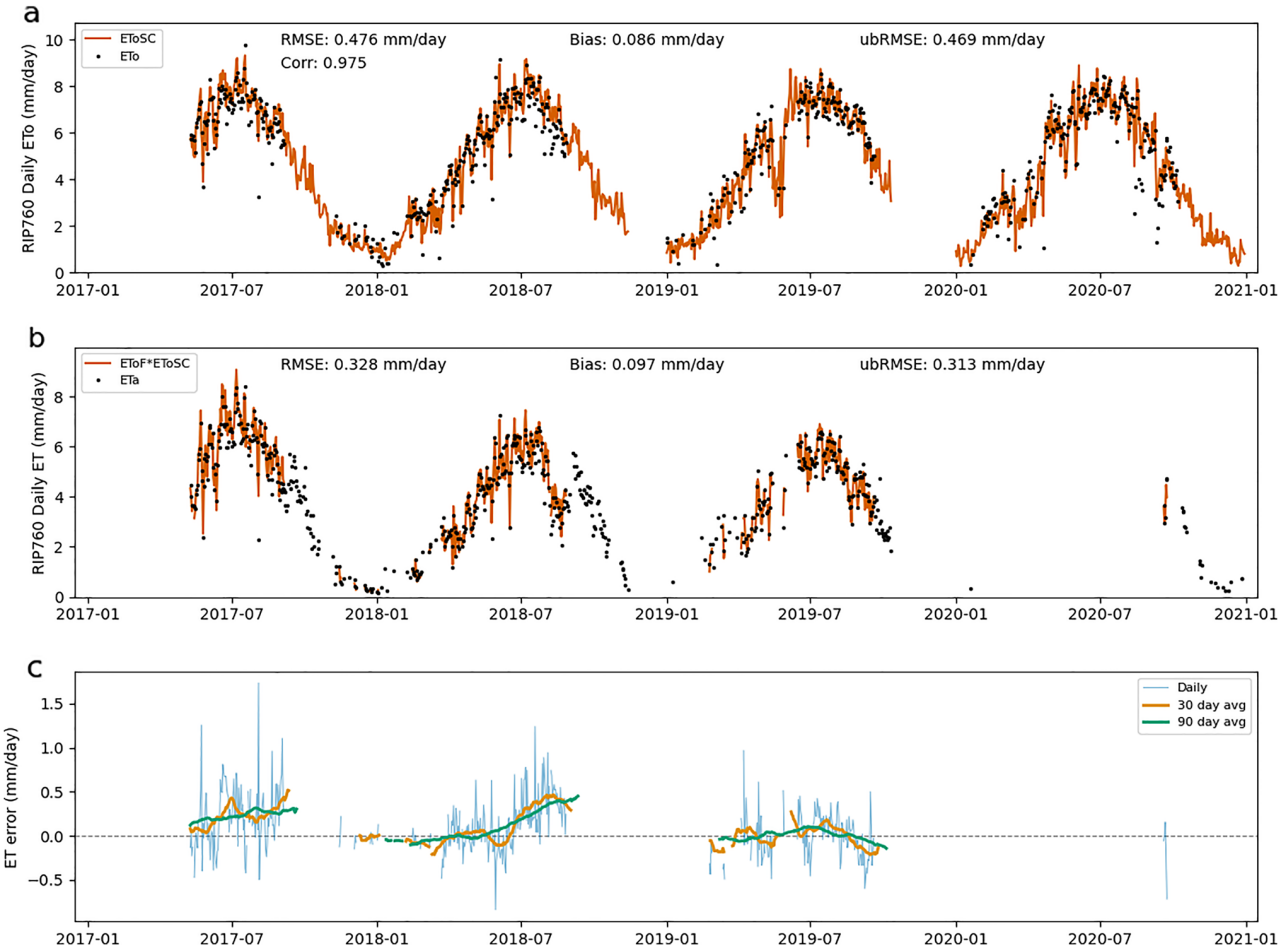
ETo errors at RIP760. Panel **a** shows EToSC versus ETo computed from in situ measurements at daily timesteps. Panel **b** shows ET computed as the product of EToSC and ground-based EToF versus ETa at daily timesteps. Panel **c** shows individual daily ET errors (Eta–EToSC*EToF) as well as averaged daily errors over 30- and 90 day windows

Errors in EToSC can lead to both systematic and random errors in ET estimates. Figures 3, 4, 5 show the time series for each site of EToSC versus ground-based ETo (Figs. 3a, 4a, 5a), ET estimated using EToSC versus ETa (Figs. 3b, 4b, 5b), and the resulting daily errors (Figs. 3c, 4c, 5c). The biases at BAR012 (Fig. 3) and SLM001 (Fig. 4) are readily apparent both in the raw data and particularly in the error time series. In addition to showing bias over the duration of the sample, we see that the expected bias for a given day appears to depend strongly on the time of year. Note that the amplitudes of the 30- and 90-day average bias regularly peak during the middle of the growing season—positive at BAR012 (Fig. 3) and negative at SLM001 (Fig. 4)—and approach zero during the winter months. This means that the effect of biases in EToSC could have a particularly significant impact on ET estimates at daily or monthly timescales. There is a similar seasonal pattern in errors at RIP760 (Fig. 5), but the amplitude of the errors is much smaller, and the bias is negligible at yearly or greater timescales.

Figures 3, 4, 5 also show the effect of random errors on overall estimates. The local (in time) variance of the errors also varies seasonally, although the pattern is less obvious than with the bias. The error variance increases slightly during the growing season, but several large daily errors are apparent in the spring and fall. Most of the random error appears to be "averaged out" in the 90-day moving average as shown by the smooth seasonal pattern in these curves. The 30-day moving averages still vary with higher frequency than the seasonal pattern shown in the 90-day. It is not immediately clear whether this variation reflects underlying structure in the errors (subtle systematic errors in EToSC) or whether the effect of the random error is still substantial at 30-day time scales.

**Fig. 6:**
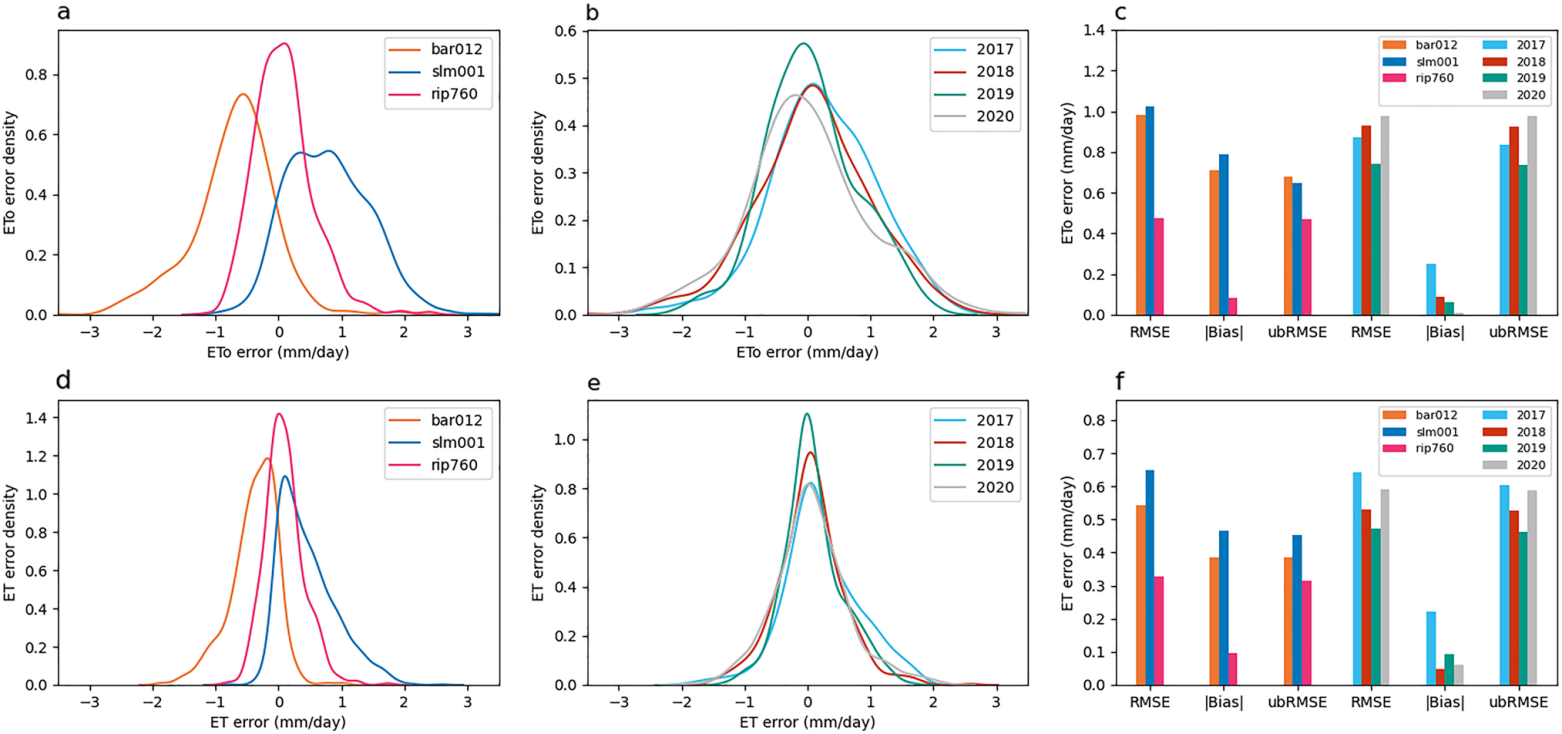
EToSC errors across sites and years. Panels **a**, **b**, **d**, and **e** are kernel density plots of errors, generated by using a Gaussian kernel. Panels **a** and **d** show the ETo and ET errors, respectively, aggregated by site. Panels **b** and **e** show the same errors aggregated by year. Panels **c** and **f** show error statistics for ETo and ET, respectively, broken out by site and by year

Despite site-level variation, some clear patterns emerge across the full data sample. Figure 6 summarizes characteristics of the errors including the distributions of errors for each site (Figs. 6a, d), the distributions of errors for each year (Figs. 6b, e), and statistics that summarize the contribution of bias and random noise to overall errors (Figs. 6c, f). Results indicate that the bias in EToSC varies in space, as demonstrated by the variation in biases at the three sites. The bias at BAR012 is negative, the bias at SLM001 is positive, and the bias at RIP760 is approximately zero (Fig. 6a and c). In contrast, the bias in EToSC does not vary significantly from year to year (Figs. 6b and c). The minor exception is 2017, when the errors skew slightly positive. This is likely a result of a seasonally biased sample, as the data records at BAR012 and RIP760 start in May 2017.

All sites and years have non-negligible random error. The amplitude of random error is estimated as ubRMSE and can be observed in the width of the error distributions. At BAR012 and SLM001, the amplitude of bias is slightly larger than the random error, but approximately equal. At RIP760, the random error is significantly larger than the bias (ubRMSE ≈ RMSE) but is still smaller than the random error at the other two sites. Over sufficiently long timescales, the impact of this random noise on time-integrated ET estimates decreases. However, the bias can also vary in time on sub-annual timescales, as shown in Figs. 3, 4, 5. This seasonally varying bias can be interpreted as noise at time scales of a year or greater.

### Error from SIMS Kcb

**Fig. 7:**
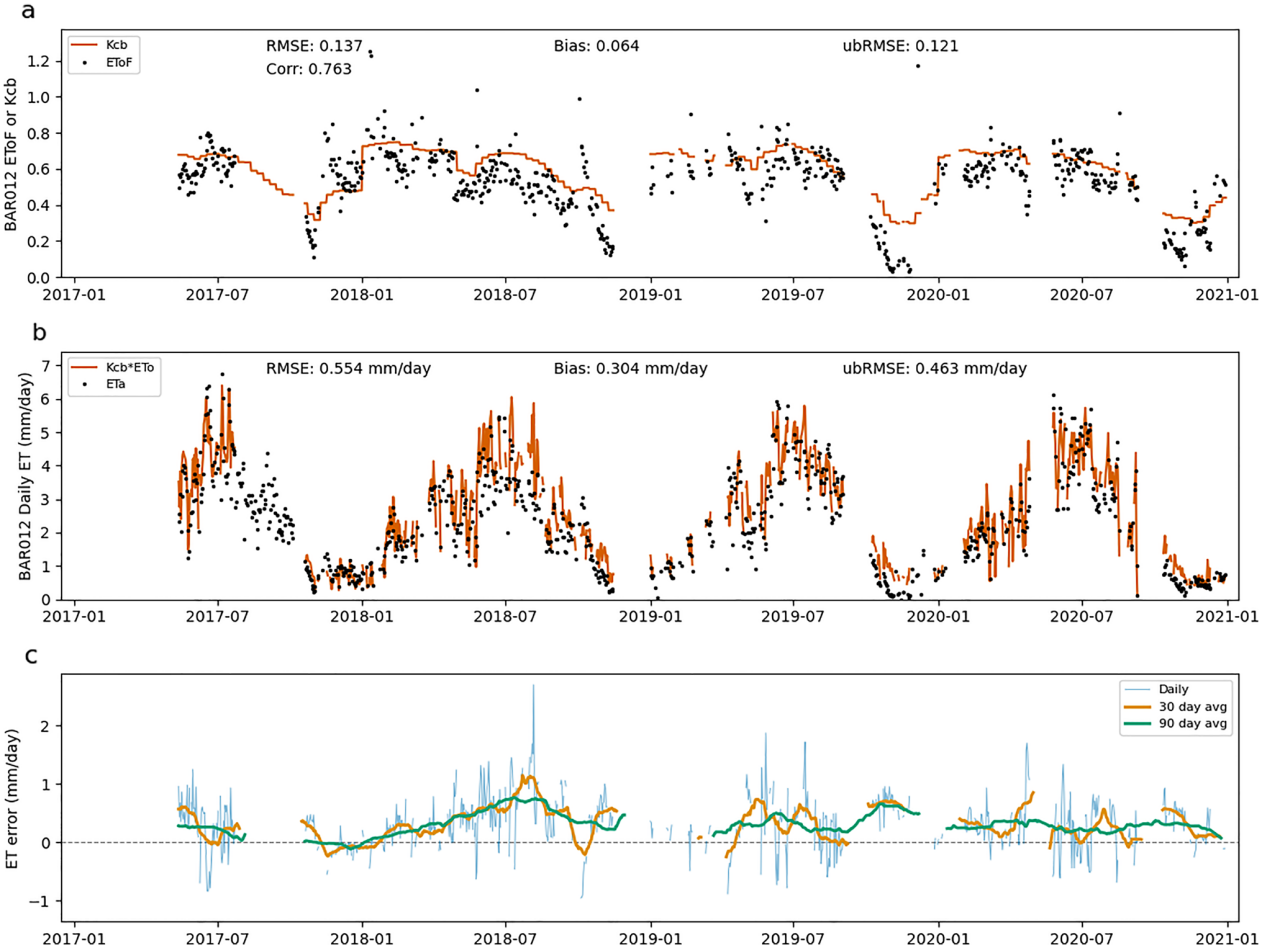
EToF errors at BAR012. Panel **a** shows SIMS Kcb versus ground-based EToF computed from in situ measurements at daily timesteps. Panel **b** shows ET computed as the product of SIMS Kcb and ground-based ETo versus ETa at daily timesteps. Panel **c** shows individual daily ET errors (ETa–ETo*Kcb) as well as averaged daily errors over 30- and 90 day windows

**Fig. 8:**
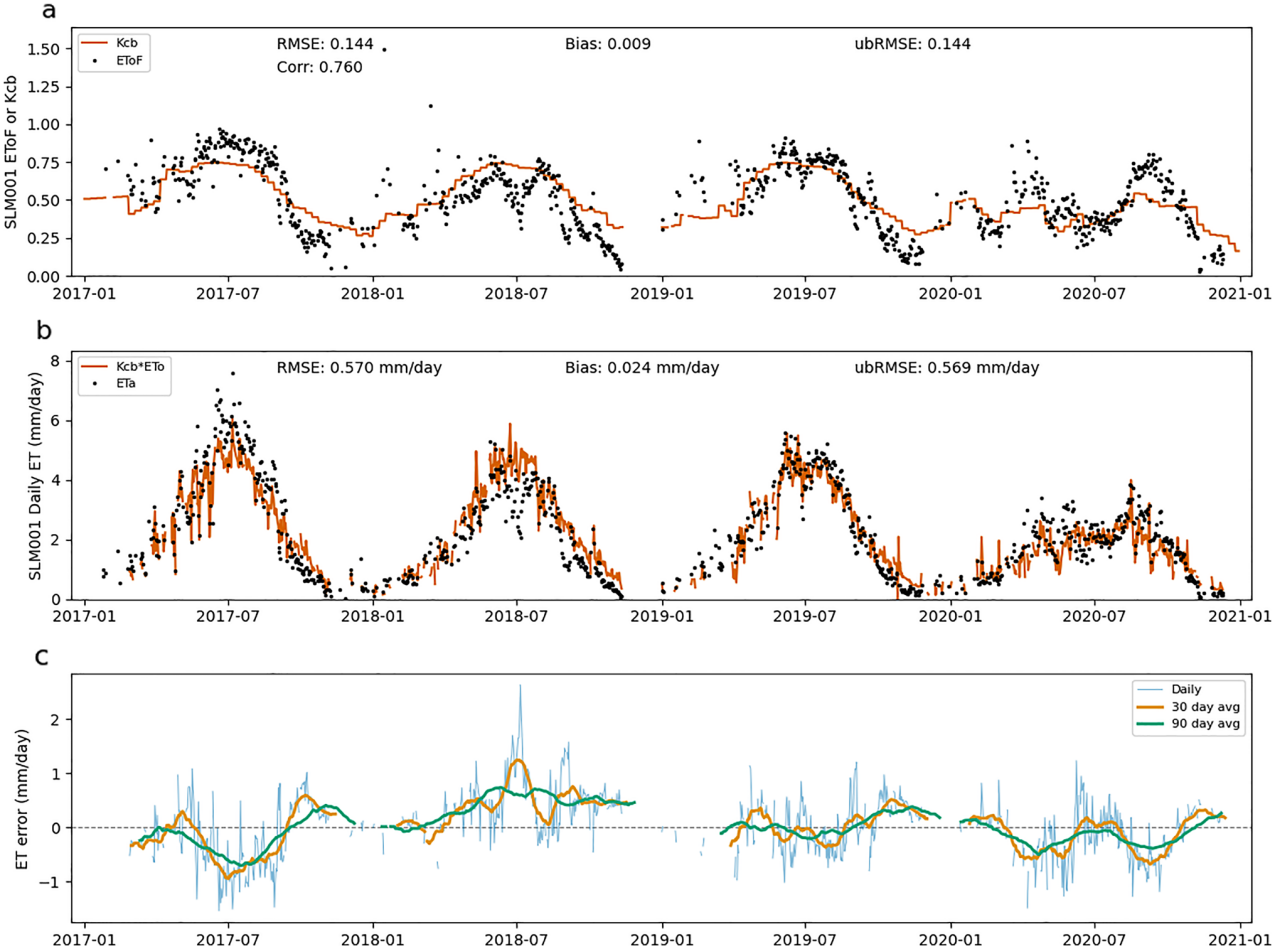
EToF errors at SLM001. Panel **a** shows SIMS Kcb versus ground-based EToF computed from in situ measurements at daily timesteps. Panel **b** shows ET computed as the product of SIMS Kcb and ground-based ETo versus ETa at daily timesteps. Panel **c** shows individual daily ET errors (ETa—ETo*Kcb) as well as averaged daily errors over 30- and 90 day windows

**Fig. 9:**
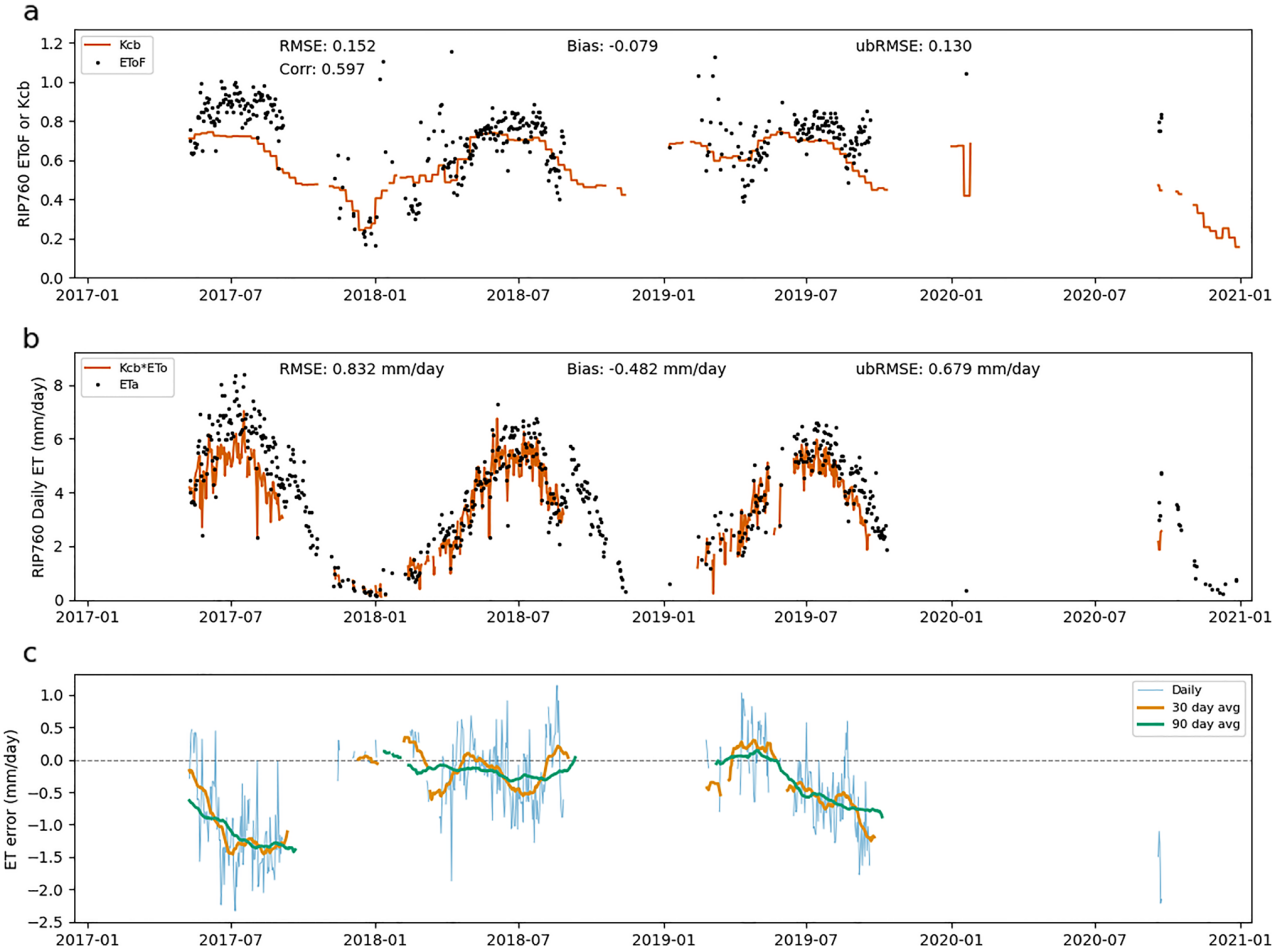
EToF errors at RIP760. Panel **a** shows SIMS Kcb versus ground-based EToF computed from in situ measurements at daily timesteps. Panel **b** shows ET computed as the product of SIMS Kcb and ground-based ETo versus ETa at daily timesteps. Panel **c** shows individual daily ET errors (ETa—ETo*Kcb) as well as averaged daily errors over 30- and 90 day windows

Errors in EToF estimates can also lead to both systematic and random errors in ET estimates. Throughout this section, "EToF errors" refers to the difference between the Kcb value calculated by SIMS and the ground-based EToF value. Figures 7, 8, 9 show the SIMS Kcb values versus EToF values (Figs. 7a, 8a, 9a), the corresponding ET values (Figs. 7b, 8b, 9b), and the daily ET errors resulting from EToF errors (Figs. 7c, 8c, 9c). The full NDVI time series, used to compute SIMS Kcb, for each site can be seen in Supplementary Fig. 1.

Figures 7, 8, 9 make it clear that Kcb errors vary seasonally and the expected error (bias) depends on time of year. At BAR012 and RIP760 (Figs. 7, 9), the (negative and positive, respectively) biases generally have the largest magnitude during mid to late summer. The magnitude of the bias varies from year to year, but the sign of the bias remains constant at these two sites. SLM001 (Fig. 8) is different in that the sign of the bias varies from year to year, with 2017 and 2020 being negative and 2018 and 2019 being positive. It is difficult to explain this variation without more information, but the simplest explanation would be differences in management practices that cause ET to be water limited (stressed) in 2018 and 2019, or for non-negligible soil evaporation from irrigation occurring in 2017 and 2020.

One source of error that is readily apparent at all three sites is the contribution of soil evaporation to overall ET. This error arises through two distinct pathways. The first is when precipitation causes the EToF to spike (often EToF > 1) before returning to a "baseline" EToF as water is quickly depleted from the evaporable zone. These precipitation spikes are visible at each of the three sites, particularly in the winter months when precipitation is more frequent and significant. While SIMS includes an optional soil water balance model to calculate soil evaporation, this function was excluded for the purposes of this analysis to focus on the errors associated solely with the satellite-based estimates of Kcb.

The second source of soil evaporation is irrigation. For example, the underestimation at RIP760 in 2017 and 2019–2020 and at SLM001 in 2017 is fairly consistent throughout the middle of the growing season when there is close to zero precipitation. The EToF stays consistently above SIMS Kcb for weeks or months, which indicates regular irrigation. The irrigation underestimates are not consistent across sites or even across years at a single site. The inter-annual variation is most easily explained by differences in management practices, particularly irrigation volume and the use of cover crops. We see an example of this at RIP760 in 2020, where the ETo is consistent with prior years but the EToF increases significantly.

There are also instances where Kcb is consistently higher than EToF, for example at BAR012 (Fig. 7) and in 2018 at SLM001 (Fig. 8). This can be caused by transpiration limitations imposed by water available in the root zone (water stress). These errors are smaller in magnitude than some of the underestimates caused by soil evaporation but can be significant when they occur in the middle of the growing season. There are cases where the hypothesized water limitation could manifest as a reduction in NDVI, which leads to a drop in Kcb. However, the degree to which transpiration is water limited likely will not be fully reflected in a drop in NDVI. This is a known fundamental limitation that SIMS, like other reflectance-based methods, can only detect chronic stress that causes reduced leaf area or fractional canopy cover, and even then it cannot detect reduction in transpiration due to transitory water limitations.

**Fig. 10:**
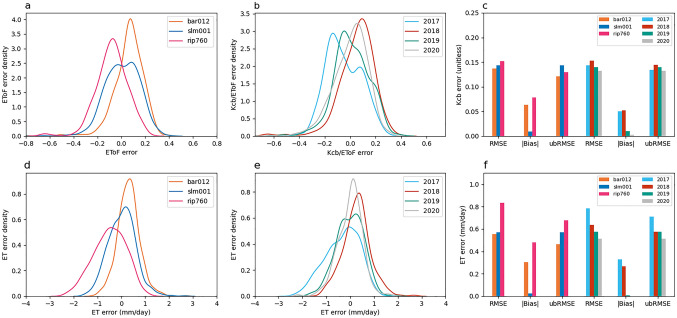
EToF errors across sites and years. Panels **a**, **b**, **d**, and **e** are kernel density plots of errors, generated by using a Gaussian kernel. Panels **a** and **d** show the ETo and ET errors, respectively, aggregated by site. Panels B and E show the same errors aggregated by year. Panels **c** and **f** show error statistics for ETo and ET, respectively, broken out by site and by year

Figure 10 summarizes characteristics of the errors in SIMS Kcb estimates and how these errors propagate to overall ET estimates. The top row (Fig. 10a, b, c) compares SIMS Kcb estimates versus the ground-based EToF values. The second row (Fig. 10d, e, f) compares ET estimated as the product of SIMS Kcb and ground ETo with ground ETa values. Like the errors from EToSC, the average bias and random error vary fairly significantly between sites. The bias at RIP760 is significant, where the satellite-driven model is underestimating the ground-based ET by more than 0.8 mm/day. This is also observed in the EToF and ET errors (Fig. 10a, b, d, and e), where the RIP760 and 2020 densities have large tails on the left side. This bias derives largely from 2020 where Kcb is significantly smaller than EToF for much of the year. In contrast, the bias is positive at BAR012 and approximately zero at SLM001 over the duration of the sample. Bias in Kcb varies from year to year more than the bias in EToF does.

At all sites and all years, ubRMSE is greater than the amplitude of the bias. On the surface, this would suggest that the effect of random error is larger than bias in estimates of Kcb/EToF. However, it is also possible that there are seasonal (non-random) errors that partially or completely counteract one another over longer timescales. This possibility merits further consideration given that errors appear to vary in multiple dimensions by site and by year. For this analysis, again we must look at the individual site time series data.

## Discussion

While there is significant variation in errors between the sites, and we cannot assume that the three vineyards included in this analysis are fully representative of all winegrape cultivation in California, our analysis reveals sources of error that are likely to affect ET estimation at many locations.

The findings on errors in spatially interpolated ETo are important not only for SIMS, but for spatially integrated ET modeling in general. Not every model uses ASCE Penman–Monteith ETo, but many spatially integrated ET models do rely on estimates of atmospheric and radiative forcing variables like temperature, humidity, and net radiation. Even with high-quality datasets like Spatial CIMIS that use an extensive network of agricultural weather stations, estimation error and representativeness error are unavoidable when using spatially gridded meteorological data. While the ETo data product explains significant temporal variation, it can have temporally persistent and spatially idiosyncratic biases due to unobserved spatial variation not captured by weather stations or extrapolated by the model.

These biases in ETo can be significant in the context of water management applications. For example, the daily biases at BAR012 and SLM001 add up to errors of –140 mm (–5.5 inches) and 170 mm (6.7 inches), respectively, when aggregated to a yearly scale. In addition, the biases appear to vary spatially as evidenced by the difference of sign at different sites. This means that addressing this source of error will require more than a spatially uniform bias correction for a given dataset. More generally, drawing strong conclusions about ET or irrigation water use will require methods for quantifying the uncertainty from spatially interpolated weather data on final ET estimates.

The errors from Kcb are harder to interpret given the nature of the comparison to EToF, but still reveal important features of SIMS and crop coefficient models generally. The first is that crop coefficient errors appear to be temporally dependent. This is not surprising, given that transpiration is partially biologically controlled and so will be related to crop phenology. Excluding site-years with significant bare-soil evaporation, Kcb tracks EToF fairly closely with some years having small positive or negative biases that might depend on management practices. However, SIMS seems to consistently overestimate EToF in winter months. This is likely due to the presence of a cover crop, which increases the NDVI (and Kcb) and can also limit bare-soil evaporation (decreases EToF). The effect of this problem is minimal on annual ET estimates due to ETo being lowest in the winter.

Table 2 summarizes some characteristics of the ET errors, including the range of approximate magnitudes across sites and year and the underlying sources of variance. The error magnitude ranges for timescales greater than a year describe ranges of errors over site and year cross sections, as depicted in Figs. 6 and 10. The 90-day bias estimates describe the ranges of 90-day moving average errors as shown in Fig. 3, 4, 5 and 7, 8, 9. The comparative assessment of sources of variance are informal due to the sample size and complexity of the data. They reflect subjective assessments of the data depicted in Figs. 6 and 10 and are not the results of rigorous statistical tests.

**Table 2:** Characterization of ET error contributions from EToSC and SIMS Kcb

Variable		Errors for timescale > 1 year			Errors for 90-day timescale	Errors for 30-day timescale
		ET RMSE	|ET bias|	ET ubRMSE	|ET bias|	|ET bias|
EToSC	Range	0.4–0.7 mm/day	0.1–0.5 mm/day	0.4–0.6 mm/day	0–1.0 mm/day	0–1.5 mm/day
	Variance	sites > time	sites > > time	time > sites	time $\approx$ sites	time $\approx$ sites
SIMS Kcb	Range	0.6–0.8 mm/day	0–0.7 mm/day	0.5–0.8 mm/day	0–2.0 mm/day	0–2.0 mm/day
	Variance	sites $\approx$ time	sites $\approx$ time	sites $\approx$ time	time > sites	time > sites

Table values synthesize information from Figs. 3–10. "Range" is the approximate range of error magnitudes calculated across sites and years. "Variance" is a comparison of the relative magnitude of error variance across sites and time. The comparison symbols, describing the relative contribution to variation in ET errors, can be interpreted as follow: "x > y" means the variation is greater in x but variation in x and y are on the same order of magnitude. "x >  > y" means most variation in errors occurs in x and that y is negligible in comparison. "x $\approx$ y" means that it is not clear that errors vary significantly more or less in x than in y.

Interpretation of the error contribution from land surface data inputs depends on application context. Throughout this study, we examine errors resulting from using SIMS Kcb as an estimate of EToF. However, for some applications, this error may be reducible. For example, in applications like irrigation scheduling where both precipitation amounts and applied water volumes and schedules are known, it is feasible to estimate the contribution of soil evaporation (Ke) and crop water stress (Ks) to the overall crop coefficient (Kc) (Allen et al. 1998). At various points in time, soil evaporation from irrigation appears to contribute significantly to errors at RIP760 (Fig. 9) and, to a lesser extent, at SLM001 (Fig. 8). In addition, intermittent periods of deficit irrigation and vine water stress are characteristic of winegrape production, and capturing this influence would be expected to further reduce the error contribution from land surface inputs and reduce the tendency of SIMS to overestimate actual ET in vineyards during the summer months, as observed at SLM001 and BAR012. In this study, we observe that the error contribution from meteorological and land surface data inputs are of similar magnitude. If applied water or soil moisture data are available and incorporated, it could make it such that the error contribution from meteorological inputs exceeds that of land surface inputs.

One common pattern across sites and years was the correlation of errors in time at sub-annual timesteps. While, on average, the ubRMSE is greater than or equal to the magnitude of bias at timesteps of a year or more, this does not mean that the majority of ET error contributions are truly random. For example, the ET estimates calculated using SIMS Kcb at SLM001 (Fig. 8) are approximately unbiased when computed over the full duration of the sample. However, the moving average errors in Fig. 8c make it clear that, for a given 30- or 90-day sample, the ET estimates may be biased. At SLM001, it happens to be the case that the time-varying local biases approximately cancel each other out over sufficiently long timescales. Time-varying local bias is present in both SIMS Kcb and EToSC. There are many potential mechanisms that could contribute to these phenomena. For SIMS Kcb, time-varying bias could arise from seasonal management practices like irrigation or the presence of cover crops. For EToSC, time-varying bias likely results from the fact that the ETo is a nonlinear function of temperature and humidity, which vary seasonally.

Improving the accuracy of the meteorological inputs can be more challenging. The most direct pathway to reducing error contributions from the meteorological inputs is to increase the density of agricultural weather station networks. Cost-effective expansion can be informed by analyses to optimize the spatial configuration of mesoscale environmental monitoring networks, such as those performed for the Kansas Mesonet (Patrignani et al. 2020). However, limitations on funding, availability of suitable locations or water supplies for irrigation of the reference crop may constrain the ability of network managers to add new sites at optimal locations. Other potential avenues for addressing error contributions include blending of meteorological inputs from mesoscale weather models, reanalysis data, and satellite observations (Pelosi et al. 2021), development of approaches to facilitate assimilation of local wind speed and other meteorological measurements collected over surfaces that differ from ASCE standard reference conditions (Anderson et al. 2017), and careful evaluation of the impacts of different spatial interpolation methods on the accuracy of the ETo data calculated from the gridded meteorological inputs (Ha et al. 2011).

In summary, this study produced results that should be of interest both to practitioners who use SIMS for irrigation scheduling, as well as for ET modeling more broadly. For the former, this study provides a robust and nuanced characterization of the errors that arise from meteorological and land surface inputs when using SIMS and other similar reflectance-based ET models. This information is useful when trying to account for uncertainty in modeled ET estimates. Regarding ET modeling more generally, the study shows that errors in meteorological forcing data can be substantial and were approximately equal to the errors from the land surface model at two out of three sites. This is important because, while it is clear how one can improve on SIMS Kcb as an estimate of EToF, the same is not necessarily true of providing meteorological inputs representative of local conditions. Data products like EToSC are generated using complex spatial curve-fitting methods, and yet still can introduce substantial error to ET estimates. Until this source of error can be reduced, it will be very important to consider its effects when applying remote sensing ET models to water resource management. Similarly, the results from Bhattarai et al. (2022) indicate that an ET model sensitive to vapor pressure deficit and wind speed requires a bias correction to weather station observations to achieve reliable daily estimates. Without local weather station observations, this will continue to be a source of error.

## Supplementary Information

Below is the link to the electronic supplementary material.

Supplementary file1 (DOCX 77 KB)
