# Supplementary material for: Effects of meteorological and land surface modeling uncertainty on errors in winegrape ET calculated with SIMS
Source: Irrig Sci. 2022 Aug 13;40(4-5):515–30. doi: 10.1007/s00271-022-00808-9 (PMC9509309; doi:10.1007/s00271-022-00808-9)
Supplement: Supplementary file 1 — Supplementary file1 (DOCX 77 KB) [file 271_2022_808_MOESM1_ESM.docx]

# 5 Supplementary information

## 5.1 HLS and interpolated NDVI time series


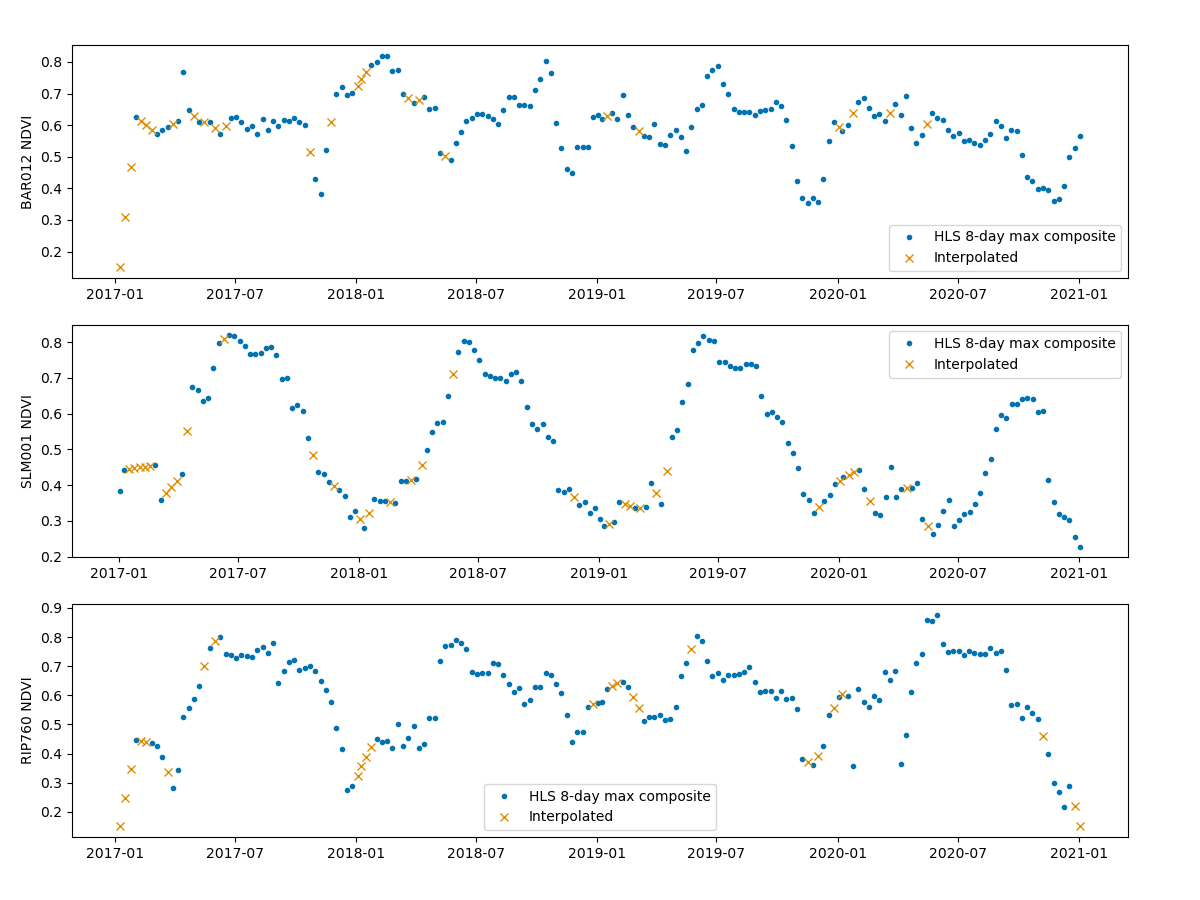


**SupplementaryFig. 1 NDVI time series for each study site.**
